# Variability in antifungal utilization among neonatal, pediatric, and adult inpatients in academic medical centers throughout the United States of America

**DOI:** 10.1186/s12879-018-3410-4

**Published:** 2018-10-03

**Authors:** Jeremy S. Stultz, Rose Kohinke, Amy L. Pakyz

**Affiliations:** 1 Department of Clinical Pharmacy and Translational Science, College of Pharmacy, The University of Tennessee Health Science Center, 881 Madison Avenue, Room 223, Memphis, TN 38163 USA; 2 Carilion Roanoke Memorial Hospital, Roanoke, VA USA; 3 School of Pharmacy, Virginia Commonwealth University, Richmond, VA USA

**Keywords:** Antimicrobial stewardship, Pediatrics, Neonates, Antifungal stewardship, Antimicrobial trends

## Abstract

**Background:**

Identification of factors associated with antifungal utilization in neonatal, pediatric, and adult patient groups is needed to guide antifungal stewardship initiatives in academic medical centers.

**Methods:**

For this hospital-level analysis, we analyzed antifungal use in hospitals across the United States of America, excluding centers only providing care for hematology/oncology patients. Analysis of variance was used to compare antifungal use between patient groups. Three multivariable linear regression models were used to determine independent factors associated with antifungal use in the neonatal, pediatric, and adult patient groups.

**Results:**

For the neonatal, pediatric, and adult patient groups, 54, 44, and 60 hospitals were included, respectively. Total antifungal use was significantly lower in the neonatal patient group (14 days of therapy (DOT)/1000 patient days (PDs) versus 76 in pediatrics and 74 in adults, *p* < 0.05). There were no significant associations identified with total antifungal DOT/1000 PDs in the neonatal patient group (model R^2^ = 0.11). In the pediatric patient group (model R^2^ = 0.55), admission to immunosuppressed service lines and total broad-spectrum antibiotic use were positively associated with total antifungal use (coefficients of 1.95 and 0.41, both *p* < 0.05). In the adult patient group (model R^2^ = 0.79), admission to immunosuppressed service lines, total invasive fungal infections, and total broad-spectrum antibiotic use were positively associated with total antifungal use (coefficients of 5.08, 5.17, and 0.137, all *p* < 0.05).

**Conclusions:**

Variability in antifungal use in the neonatal group could not be explained well, whereas factors were associated with antifungal use in the adult and pediatric patient groups. These data can help guide antifungal stewardship initiatives.

## Background

Invasive fungal infections (IFIs) are recognized complications that can cause significant morbidity and mortality in immunosuppressed and critically ill neonatal, pediatric and adult patients [1–3]. Similar to antibiotics, frequent use of common antifungal agents has been associated with increased resistance in adults, which could pose a challenge to the effective management of IFIs [4, 5]. Recent trends have suggested there are increasing rates of non-*Candida albicans* species causing IFIs, which may have more resistance to the commonly used antifungal, fluconazole [3]. Appropriate and judicious use of antifungals may help preserve the utility of currently available antifungals in treating IFIs. In the 2016 Infectious Diseases Society of America (IDSA)/ Society for Healthcare Epidemiology of America (SHEA) recommendations regarding Antimicrobial Stewardship (ASP) implementation, ASP interventions for immunocompromised patients receiving antifungal therapy are recommended [6]. This recommendation was based on a low quality of evidence.

Published antifungal stewardship interventions have included activities such as prior authorization, restrictions, and prospective audits with feedback. Most of these studies occurred at single institutions [5, 7–10]. Additional studies are needed to guide antifungal stewardship practices across multiple institutions. Antifungal usage trends and factors associated with use in United States of America (US) academic medical centers (AMCs) have been described in adults [11]. This prior study utilized data from 2004 to 2008 and antifungal utilization and antifungal stewardship practices may have changed since then, necessitating additional analysis.

The pediatric and neonatal populations have unique ASP needs and IFI etiology, which will alter the needs of age-specific antifungal stewardship initiatives [1, 12, 13]. The IDSA/SHEA ASP implementation guidelines suggest that although ASP activities may not have been studied to the same extent in the pediatric population compared to the adult population, the activities are likely effective and should be implemented. The guidelines also recommend antibiotic stewardship in neonates, but do not mention antifungal stewardship in this setting [6]. Additional analysis is needed to effectively construct ASP and antifungal stewardship activities in the pediatric population. Freestanding children’s hospitals may have differences in antifungal use and/or ASP practices compared to an AMC (i.e., a pediatric hospital housed within an adult hospital) where pediatric and neonatal policies are more likely to be extensions of adult policies. Trends in antifungal use over time have been previously described among freestanding pediatric US institutions, but not in pediatric hospitals within an AMC [14]. Additionally, factors associated with antifungal use have not been identified in the pediatric population. Thus, there is limited data in AMCs to determine antifungal usage patterns and guide potential pediatric and neonatal antifungal stewardship activities.

The objectives of our study of AMCs across the US were multifold. First, we wanted to describe antifungal use in the neonatal, pediatric, and adult patient groups. Second, we aimed to identify antifungal stewardship targets by evaluating factors associated with antifungal use in neonatal, pediatric, and adult patients. As a tertiary aim, we sought to identify if antifungal restriction impacted antifungal use.

## Methods

### Hospital data source

The data for this hospital-level cross-sectional analysis were obtained from Vizient, an alliance of US hospitals, inclusive of AMCs. Data were obtained for AMCs from the Clinical Data Base Resource Manager (CDB/RM) of Vizient. The CDB/RM includes charge-based inpatient medication, procedure, and diagnosis specific data. Data obtained from the CDB/RM have been utilized in previous studies regarding antimicrobial use [11, 15]. Vizient hospitals are located in all major US geographic regions. Information was collected from Vizient members that consistently reported data from January 1, 2015 through December 31, 2015.

### Data collection

Data were collected for three separate patient groups: adult inpatients (≥18 years), pediatric inpatients (< 18 years excluding the neonatology service), and neonates/infants classified under the neonatal service line. Normal newborns were excluded from the analysis due to lack of antifungal use. Centers only providing care for hematology/oncology patients (*n* = 5) and those hospitals with no reported use of any target agents were excluded.

Within each identified patient group, systemic antifungal use was measured for azoles (fluconazole, itraconazole, voriconazole, and posaconazole), echinocandins (caspofungin, micafungin, and anidulafungin), and polyenes (amphotericin B deoxycholate, liposomal amphotericin B, and amphotericin B lipid complex). Agents that are only intended for topical use or only as combination therapy (i.e., flucytosine) were excluded. Usage was expressed as days of therapy (DOT) normalized per 1000 patient-days (PD). One DOT represented the receipt of an antifungal agent on a given day, regardless of the strength, number of doses, or route of administration. Average length of therapy (LOT) was also determined in days, but was not normalized since patient level data was not available.

Potential factors associated with antifungal use were measured for the three patient groups from the CDB/RM. The frequency of International Classification of Diseases version 9 (ICD-9) codes for an IFI was measured as admissions/1000 PD. All relevant 112, 114, 115, 116, and 117 codes excluding mucosal or superficial infections were included. The number of DOT/1000 PD of broad-spectrum antibiotics (defined as carbapenems, intravenous third- or fourth-generation cephalosporins, and antipseudomonal β-lactam/ β-lactamase inhibitor combinations and fluoroquinolones) was measured, as antibiotics have served as an independent risk factor for candidemia [16, 17]. Geographic location of the institution was collected (i.e., Northeast, South, Midwest, West). Data concerning clinical service line (CSL) distributions within the pediatric and adult patient groups were obtained and measured as PD admitted to the service line/1000 PD. The 32 different Vizient CSLs were collapsed into four categories: medicine; surgery; and immunosuppressed (e.g., oncology, transplant); and other. The number of extremely low birthweight neonates, defined as a birthweight of < 1000 g, was collected for the neonatal patient group since fluconazole prophylaxis may be considered in these patients [18]. To account for the level of neonatal care provided, institutions with at least one extracorporeal membrane oxygenation (ECMO) case in 2015 were categorized as ECMO centers. The neonatal intensive care unit levels 1–4 were not used as variation in level definition occurs between states. Finally, data concerning the extent of ASP involvement at the institution were obtained from a previously administered survey concerning ASPs as described in the following section.

### Antimicrobial stewardship questionnaire

A survey requesting information regarding antimicrobial stewardship practices was sent via email in March 2016 to Vizient members through the Vizient antimicrobial stewardship list-serve. Two questions from this survey were used for this study. One question was utilized as a possible factor related to antifungal use. It asked institutions how often they performed post-prescription ntibiotic review across seven different adult hospital service line categories and in pediatric and neonatal intensive care unit (NICU) populations. The answer options were: always/almost always; more than half the time; one-quarter of the time; not currently performed; and not applicable. This question was included to approximate the extent of an institution’s ASP. For analytical purposes, institutions were categorized as having extensive ASP involvement or not. In the adult analysis, extensive ASP involvement was defined as performing post-prescription review half of the time or more in five main service lines (medical intensive care unit (ICU), surgical ICU, surgical units, non-ICU medical floors, and oncology). The other two service lines (obstetrics/gynecology and rehabilitation) were not included in the definition due to low ASP activities being performed across institutions in those areas. In the pediatric and neonatal analysis, any response of an institution performing pediatric or neonatal post-prescription review half the time or more was considered extensive.

A second survey question was included to help achieve our tertiary study objective. It asked if an echinocandin, usually caspofungin or micafungin, was on the hospital’s formulary and if use was restricted. Antifungal use data were compiled based on echinocandin restriction for comparison. Hospitals were excluded from this analysis if they did not answer this survey question or did not have a matching hospital that fulfilled other inclusion criteria. This question was not included as a potential factor for antifungal use, because the survey question did not specify to which of the patient groups the restrictions applied.

### Statistical analysis

Descriptive statistics utilized were mean with standard deviation (SD) or 95% confidence interval. Analysis of variance with post-hoc Tukey analysis was performed to compare antifungal usage trends between the three patient groups. To identify the factors associated with total antifungal use for each patient group we performed three linear regression analyses, one for each patient group. Univariable linear regression was performed on all potential explanatory factors collected as part of this study and any factors with a significance level of < 0.25 on univariable analysis were then included in a multivariable linear regression model. Variance inflation factors were evaluated to assess for collinearity. For antifungal use comparisons between hospitals restricting echinocandins and those not restricting echinocandins, the T-test or Wilcoxon rank sum test was used based on data normality and sample size. All tests were two-tailed when applicable and the significance level was set at 0.05. All statistical tests were performed using JMP software (JMP Pro 13; SAS Institute, Inc., Cary, NC) with the exclusion of regression analyses, for which STATA software (version 13; Stata Corporation, College Station, TX) was used.

## Results

### Hospital characteristics

Complete medication data for 2015 and survey responses were available for 54, 44, and 60 of the hospitals for neonatal, pediatric, and adult patient groups, respectively. This represented over 2.1 million patient admissions. Table 1 provides additional details regarding hospital characteristics based on patient group. Medicine service lines represented the most patient days in the pediatric and adult population, and broad-spectrum antibiotic use was higher in the pediatric and adult patient groups compared to the neonatal population. Adults represented the largest patient group based on admissions and had the most IFIs based on ICD-9 codes. For over 50% of the surveyed institutions, ASP activity was considered extensive (Table 1).

**Table 1:** Characteristics of hospitals based on patient group

Characteristic	Neonates: *n* = 54; 100,155 admissions	Pediatrics: *n* = 44; 159,936 admissions	Adults: *n* = 60; 1,841,667 admissions
Geographic region, no. (%)			
South	17 (31)	15 (34)	17 (28)
Midwest	14 (26)	12 (27)	17 (28)
Northeast	13 (24)	9 (21)	13 (22)
West	10 (19)	8 (18)	13 (22)
CSL mean PD/1000 PD (SD)			
Medicine	NA	132 (55)	73 (17)
Surgery	NA	72 (27)	57 (13)
Immunosuppressed	NA	18 (9)	12 (4)
Other	NA	35 (43)	31 (13)
Patient mix, mean admissions/1000 PD (SD)			
Total invasive fungal infections	0.5 (0.9)	0.8 (1.4)	4.7 (2.6)
Extremely low birthweight	19 (18)	N/A	N/A
Patient mix, mean % (SD)			
Group admissions per total hospital admissions	5 (1.5)	10 (5)	89 (10)
Broad spectrum antibiotic use, DOT/1000 PD (SD)	84 (97)	273 (90)	283 (55)
Extensive^a^ ASP, no. (%)	36 (67)	26 (59)	32 (53)

*CSL* clinical service line, *PD* patient days, *DOT* days of therapy, *ASP* antimicrobial stewardship program

^a^ Defined for adult group as performing ASP half of the time or more in 5 main service lines (medical intensive care unit (ICU), surgical ICU, surgical units, non-ICU medical floors, and oncology). For pediatric and neonatal groups, any response of an institution performing pediatric or neonatal ASP half the time or more was considered extensive ASP activities

### Antifungal use among patient groups

Table 2 provides details regarding antifungal use for each of the patient groups. The most commonly used antifungal in all patient groups was fluconazole, which encompassed 85% of antifungal use in the neonatal group. Voriconazole and micafungin were the next most common antifungals used in the pediatric and adult group (13–16% of use in these two groups), although both agents had less use in the neonatal group (Table 2). For all azole antifungals, the mean (SD) LOTs were 7.6 (3.3), 7.7 (2.9), and 6.5 (1.5) days for the neonatal, pediatric, and adult groups, respectively (*p* = 0.0383). The mean (SD) LOTs for amphotericin B products were 7.8 (8.9), 12.3 (8.7), and 7.5 (2.6) days for the neonatal, pediatric, and adult groups, respectively (*p* = 0.0118). Post-hoc Tukey analysis suggested the pediatric patient group had longer LOT than both the neonatal and adult group (both *p* < 0.05). The mean (SD) LOTs for the echinocandin class were 13.9 (16), 12.5 (5.1), and 8 (1.6) days for the neonatal, pediatric, and adult groups, respectively (*p* = 0.0014). Post-hoc Tukey analysis suggested the adult group had significantly shorter LOT than the neonatal and pediatric groups (both *p* < 0.05).

**Table 2:** Antifungal use among hospitals based on patient group

Antifungal agent	Neonates: *n =* 54; 100,155 admissions	Pediatrics: *n =* 44; 159,936 admissions	Adults: *n =* 60; 1,841,667 admissions	ANOVA (F-statistic)
Total antifungals^*^	14.4 (10.7 to 18.2)	75.8 (56.6 to 95.1)	73.2 (62.7 to 84.4)	*p* < 0.001 (34.8)
Azoles^*^	12.3 (8.6 to 15.6)	54.6 (40.8 to 68.4)	55.1 (47.2 to 63.4)	*p* < 0.001 (32.7)
Fluconazole	12.2 (8.8 to 15.6)	40.6 (31.7 to 49.4)	37.9 (33.3 to 42.4)	
Voriconazole	0.03 (− 0.03 to 0.09)	12.5 (6 to 19)	10 (7.8 to 12.3)	
Posaconazole	0	0.91 (− 0.02 to 1.8)	5.8 (3.4 to 8.3)	
Itraconazole	0	0.61 (0.12 to 1.1)	1.3 (0.58 to 2.1)	
Amphotericin B^*^	1.4 (0.71 to 2.1)	3.8 (1.9 to 5.7)	3.9 (3.0 to 4.8)	*p* = 0.0021 (6.1)
Conventional	0.66 (0.23 to 1.1)	0.78 (− 0.07 to 1.6)	1.3 (0.76 to 1.9)	
Liposomal	0.25 (0.03 to 0.47)	2.3 (0.72 to 3.9)	2.1 (1.5 to 2.6)	
Lipid complex	0.5 (−0.018 to 1.02)	0.72 (− 0.002 to 1.43)	0.5 (0.24 to 0.78)	
Echinocandins^*^	0.76 (0.32 to 1.2)	17.4 (11.4 to 23.5)	14.3 (11.8 to 16.8)	*p* < 0.001 (28)
Caspofungin	0.17 (−0.005 to 0.34)	4.9 (0.5 to 9.3)	2.9 (1.2 to 4.6)	
Micafungin	0.59 (0.21 to 0.98)	12.5 (7.3 to 17.8)	10.4 (7.9 to 12.9)	
Anidulafungin	0	0	1 (0.4 to 1.7)	

ANOVA, analysis of variance

All data presented as mean (95% CI) days of therapy/1000 patient days

*Mean days of therapy/1000 patient days was statistically different between the neonatal group and the adult and pediatric group on post-hoc Tukey analysis, all *p* < 0.01, no significant differences occurred between the pediatric and adult groups, all *p* > 0.12

There were significant differences in the mean total antifungal use (expressed as DOT/1000 PD) and class-specific antifungal use among the patient groups. Based on post-hoc Tukey analysis, the antifungal DOT/1000 PD means were generally the same between the adult and pediatric patient groups, and significantly less in the neonatal group compared to adults and pediatrics (all *p* < 0.007, Table 2).

### Factors related to antifungal use

Table 3 provides details regarding the univariable and multivariable linear regression analyses. The neonatal group model did not explain well the variation in antifungal use between institutions (R^2^ = 0.11) despite utilizing the log DOT/1000 PD, while the adult and pediatric patient group models respectively explained 55% and 79% of the variation. In the neonatal patient group, total IFIs, being an ECMO center, and broad-spectrum antibiotic use had significant positive associations with antifungal use on univariable analysis, although none of these factors had significant associations on multivariable analysis. The pediatric patient group had significant negative associations with antifungal use in medicine and surgical service lines and positive associations for immunosuppressed service lines and total broad-spectrum antibiotic use upon multivariable analysis (all *p* < 0.05). The associations with surgery service lines was only found on multivariable analysis. The adult patient group had significant and positive correlations with antifungal use and immunosuppressed service lines, total IFIs, and total broad-spectrum antibiotic use (all *p* < 0.015).

**Table 3:** Linear regression models for factors associated with antifungal use

Variable	Univariable			Multivariable		
	Coefficient (95% CI)	Standard error	*p*-value	Coefficient (95% CI)	Standard error	*p*-value
Neonates (R^2^ adjusted =0.11)^a^: *n* = 54 institutions; 100,155 admissions						
ELBW admissions	0.016 (− 0.002 to 0.035)	0.009	0.083	−0.011 (− 0.039 to 0.016)	0.014	0.418
Total invasive fungal infections	0.442 (0.107 to 0.777)	0.167	0.011	0.375 (−0.185 to 0.935)	0.279	0.185
Total broad-spectrum antibiotics	0.005 (0.001 to 0.009)	0.002	0.012	0.002 (−0.003 to 0.007)	0.002	0.414
Extracorporeal membrane oxygenation center	0.899 (0.275 to 1.52)	0.311	0.006	0.494 (− 0.305 to 1.29)	0.397	0.220
Region (referent group = Northeast)	–			–		
South	0.864 (−0.016 to 1.75)	0.438	0.054	0.278 (−0.743 to 1.30)	0.507	0.587
Midwest	0.522 (−0.399 to 1.44)	0.458	0.260	0.391 (−0.54 to 1.32)	0.462	0.402
West	0.006 (−0.999 to 1.01)	0.500	0.991	−0.256 (1.25 to 0.737)	0.493	0.606
Pediatrics (R^2^ adjusted =0.55): *n* = 44 institutions; 159,936 admissions						
Medicine service lines admissions	−0.35 (− 0.69 to − 0.01)	0.168	0.044	−0.35 (− 0.658 to-0.045)	0.151	**0.026**
Immunosuppressed service lines admissions	2.08 (0.055 to 4.1)	1.00	0.044	1.95 (0.061 to 3.84)	0.932	**0.043**
Surgery service lines	−0.573 (−1.27 to 0.12)	0.345	0.104	−1.05 (−1.75 to − 0.345)	0.347	**0.005**
Total invasive fungal infections	10.1 (−3.54 to 23.7)	6.76	0.142	1.75 (−8.62 to 12.1)	5.11	0.733
Total broad-spectrum antibiotic use	0.327 (0.135 to 0.521)	0.095	0.001	0.41 (0.254 to 0.572)	0.075	**< 0.001**
Region (referent group = Northeast)	–			–		
South	21.2 (−32 to 75)	26.6	0.430	−11.9 (−53.9 to 30)	20.6	0.568
Midwest	47.2 (−9.1 to 103)	27.8	0.099	23.7 (−19.3 to 66.7)	21.1	0.279
West	34.8 (−27 to 97)	30.7	0.264	42.6 (−2.27 to 87.5)	22.1	0.062
Adults (R^2^ adjusted =0.79): *n* = 60 institutions; 1,841,667 admissions						
Medicine service lines admissions	−1.255 (−1.8 -to −0.70)	0.27	< 0.001	−0.311 (−0.724 to 0.102)	0.21	0.137
Immunosuppressed service lines admissions	8.03 (8.03 to 9.61)	0.79	< 0.001	5.08 (3.56 to 6.6)	0.76	**< 0.001**
Total invasive fungal infections	12.4 (9.65 to 15.2)	1.34	< 0.001	5.17 (1.82 to 8.52)	1.7	**0.003**
Total broad-spectrum antibiotics	0.345 (0.167 to 0.523)	0.89	< 0.001	0.137 (0.032 to 0.242)	0.05	**0.011**
Extensive^b^ ASP (referent = not extensive ASP)	21.4 (−0.263 to 43)	10.8	0.053	2.75 (−8.14 to 13.6)	5.4	0.614
Region (referent group = Northeast)	–			–		
South	9.12 (−21.4 to 39.7)	15.3	0.552	−9.65 (− 24 to 5.4)	7.5	0.204
Midwest	12.2 (−18.3 to 42.8)	15.3	0.425	2.25 (−12.8 to 17.3)	7.5	0.764
West	33.9 (1.38 to 66.5)	16.2	0.041	4.84 (−12 to 22)	5.4	0.569

*ELBW* extremely low birthweight < 1000 g, *ASP* antimicrobial stewardship program

Data normalized as admissions/1000 patient admissions or days of therapy/1000 patient days when appropriate

Table only includes variables with *p* < 0.25 on univariable analysis

^a^ log days of therapy/1000 patient days was utilized

^b^ Defined for adult group as performing ASP half of the time or more in 5 main service lines (medical intensive care unit (ICU), surgical ICU, surgical units, non-ICU medical floors, and oncology). For pediatric and neonatal groups, any response of an institution performing pediatric or neonatal ASP half the time or more was considered extensive ASP activities

Bolded *p*-values were statistically significant

### Impact of Echinocandin restriction

Table 4 compares the institutions overall that restricted echinocandin use on their hospital formulary with hospitals that did not restrict echinocandin use. When including all three patient groups, there was no difference in the antifungal utilization or total IFIs. If only the adult patient group was examined, there was a significantly higher use of all antifungals at institutions that restricted echinocandins (*p* < 0.001) and no difference in total IFIs.

**Table 4:** Antifungal use among overall patient groups and the adult group by hospital echinocandin restriction

Agent	Overall			Adults		
	Restriction*: n =* 43	No Restriction: *n =* 17	*p-*value (t-ratio or Z-statistic)	Restriction: *n =* 43	No Restriction: *n =* 17	*p-*value (t-ratio)
Fluconazole	29.4 (24.5 to 34.3)	28.8 (22.5 to 35)	0.16^a^ (0.48)	39 (34 to 44.2)	36 (24.2 to 45.2)	0.15^b^ (0.8)
Echinocandins	10.9 (8 to 14)	8.6 (4.8 to 12.4)	0.34^a^ (−0.9)	15 (12 to 18.4)	12 (8.5 to 15.7)	0.41^b^ (1.3)
All Antifungals	53.7 (43.1 to 64.4)	49.7 (35.8 to 63.5)	0.3^a^ (−0.08)	76 (63.6 to 89)	66 (44 to 88.4)	**< 0.001**^b^ (**0.83)**
Invasive Fungal Infections	0.72 (0.55 to 0.9)	0.65 (0.53 to 0.77)	0.538^b^ (1.41)	0.81 (0.65 to 0.97)	0.72 (0.57 to 0.86)	0.3934^b^ (0.57)

Antifungal data presented as mean (95% CI) days of therapy/1000 patient days

Invasive fungal infections data presented as mean (SD) admissions/1000 patient days

^a^ Wilcoxon rank sum compared hospitals restricting echinocandins to those without restrictions

^b^ T-test compared hospitals restricting echinocandins to those without restrictions

Bolded *p*-values were statistically significant

## Discussion

This study provides a unique analysis of antifungal utilization within AMCs in the neonatal, pediatric, and adult patient groups and identified factors associated with antifungal use. These data can help guide antifungal stewardship initiatives in the pediatric and neonatal patient groups at AMCs where such programs may be less frequently implemented.

These data showed that fluconazole remained the most commonly used antifungal in 2015 at the included hospitals among all patient groups, followed by voriconazole and micafungin in the adult and pediatric groups. Conventional amphotericin B was the second most highly used antifungal in the neonatal group, although closely followed by micafungin. Compared to a study from the same database performed in 2004–2008, adult utilization was similar except for a decrease in overall amphotericin use (5.1 DOT/1000 PD in 2008 to 3.9 in 2015), an increase in echinocandin use with a switch from caspofungin predominance to micafungin predominance (13.1 DOT/1000 PD to 14.3), and a decrease in voriconazole use (14 DOT/1000 PD to 10) [11]. Recent analyses using a different database have shown a similar trend of increasing echinocandin usage since 2006 in adults [19].

In a previous study of US freestanding children’s hospitals from 2000 to 2006, echinocandin percent utilization was the same or less than amphotericin B products, itraconazole was minimally used and posaconazole suspension was not FDA approved until 2006 [14]. Echinocandins were used more in our study in the pediatric population than amphotericin B products and posaconazole and itraconazole did have some use. These data suggest antifungal use is changing away from amphotericin B products compared to previous studies in the pediatric and adult patient groups. In the neonatal patient group, there was minimal use of echinocandins in 2006 in the previous study in freestanding children’s hospitals and while our study did have greater echinocandin use, amphotericin B still had higher use [14]. It is important to note that our study had different methodology and there was a 10-year difference between our study and prior studies. While not all antifungals are currently approved by the US Food and Drug administration for use in neonatal and pediatric patients, some changes in FDA approval status (e.g., caspofungin) and increased clinical studies and experience over the 10-year timeframe could explain some of the differences in antifungal use. Additionally, different study methodologies and possible differences between freestanding children’s hospitals and AMCs could explain differences between these studies. A future study could aim to compare use in freestanding hospitals to AMCs during similar timeframes.

A recent analysis of antifungal usage in 2015 among US freestanding children’s hospitals also suggested that echinocandin use was higher than amphotericin B [20]. This study did not separate the neonatal population and only included the oncology, bone marrow transplant, and solid organ transplant population. In 2012, a single day point prevalence study completed in 226 European centers found opposing results compared to studies, including ours, from the US. When combining neonate and pediatric patients in this study, conventional amphotericin B was the second most utilized medication at 30% of the prescribed medications (only 5–8% of use in our study for pediatric and neonatal patient groups) and echinocandins were only used 10% of the time [21]. This suggests there are likely differing practices between countries regarding antifungal use in addition to variations within a country as noted in our study and other analyses [20].

The pediatric and neonatal groups generally had longer LOT compared to adult groups, with notable differences of about 3–5 days for the echinocandin class. It was not possible from this data to determine why this occurred and this could be an area for additional research. Stewardship programs reviewing antifungal use in pediatric patients may want to focus on assessment of appropriate treatment durations.

There were some interesting differences among the factors associated with antifungal use between the pediatric and adult populations. Analysis of associated factors for antibiotic and antifungal use has been performed previously in adults to identify ASP targets [11, 15], but in the pediatric and neonatal populations similar analyses have only been completed for antibiotics [5, 22]. Our study did not identify any associations with use in the neonatal group on multivariable analysis (Table 3). Invasive fungal infections determined by ICD-9 codes were not associated with antifungal use in this group, which suggests either ICD-9 codes are not accurate in these patient groups or that most of the use in this population is for empiric therapy or prophylaxis [18]. Our inability to identify any factors could also be a result of not including other clinical factors (e.g., central line access, parenteral nutrition, corticosteroid use). However, extremely low birthweight was included and this factor is the main reason to consider antifungal prophylaxis in the neonatal population based on the IDSA guideline [18, 23].

Our results in the neonatal patient group most likely suggest that there are wide practice variations among pediatric hospitals within AMCs, which has been described for antibiotic use in children and in other patient populations receiving antifungals [5, 24, 25]. The large variability noted in neonatal antifungal practices despite controlling for multiple possible factors highlights that AMC stewardship programs should consider reviewing these populations to ensure use is appropriate. Due to the likely low number of cases, this could be an area for review of appropriateness on a daily basis. The IDSA ASP guidelines do not specify whether antifungal stewardship should be employed in the NICU population [6], but our study suggests antifungal in addition to antimicrobial stewardship is needed in AMC NICUs.

The pediatric and adult patient groups had more identified factors for antifungal utilization (Table 3). This suggests more consistent practices exist between institutions in these patient groups, despite higher use. Invasive fungal infections based on ICD-9 codes had a positive association in the adult populations and suggests treatment for fungal infections was more common in this population. The association between broad-spectrum antibiotic use and antifungal use identified in the pediatric and adult groups has been previously described and is of concern [11]. It is possible this finding is only an identified factor due to disease severity instead of causation and more analysis is needed. Identification of immunosuppressed service lines as an associated factor for antifungal use in the pediatric and adult groups suggests institutions should review antifungal regimens among these patients to ensure appropriate use. Guideline development, periodic utilization reviews, or antifungal restrictions may be more feasible stewardship initiatives versus daily review due to a higher volume of use among the immunosuppressed.

In our study, we sought to determine if there was a link between the extent of ASP involvement and antifungal use by two different analyses. The reported use of post-prescription review more than half of the time in multiple locations within the hospital (or at all in the pediatric and neonatal groups) was included in our linear regression models, but was not found to be associated with antifungal use in any of the patient groups. This suggests that ASP programs are likely not yet incorporating antifungals into their post-prescription reviews, they are not implemented effectively, and/or this marker was not an accurate reflection of the extent of ASP and antifungal stewardship practices. Formulary restriction is another recommended activity for an ASP and our exploratory analysis regarding the effect of restriction on antifungal use provided interesting insights. Institutions that restricted echinocandins had significantly higher total antifungal utilization in the adult patient group despite a similar number of IFIs. This was an unexpected finding and deserves additional analysis to identify possible causes. Some restrictions may be in place due to cost versus based on appropriate usage. Additionally, the difference was not noted in overall patient groups after including the pediatric and neonatal population, which may suggest differences in usage policies between the neonatal, pediatric, and adult populations within the included hospitals. Further analysis regarding the effectiveness and reasons behind antifungal stewardship restrictions is needed.

Our study has some limitations. The data analyzed were hospital-level and thus details of the indications for antifungal use could not be readily obtained. However, hospital-level data has the advantage of looking at a larger population of patients at multiple centers and has been used in previous studies [11, 22]. The use of ICD-9 codes for IFIs may not be the most accurate means of identifying all patients with fungal infections, although their designation was associated with use in the adult group. The survey questions may not have accurately reflected ASP involvement at an institution, although this may be the only means by which this data can be acquired. An exact survey response rate could not be calculated because the survey was sent out to multiple people at the same institution and not all hospitals who received the survey had completed 2015 antifungal data. Importantly, only unique hospital responses were included for analysis. We excluded hospitals only providing care for hematology/oncology patients to ensure a more homogeneous set of institutions, but antifungal use is likely different in these institutions and this serves as an area for future analysis.

## Conclusions

In AMCs, there are differences in antifungal utilization between neonatal, pediatric, and adult patient groups. Factors associated with antifungal use were not able to be identified for the neonatal patient group and this suggested a wide variation in practices due to unknown factors and a need for antifungal stewardship in this area. In the pediatric and adult patient groups admission to immunosuppressed service lines and broad-spectrum antibiotic use were positive factors associated with antifungal use. Review of antibacterial and antifungal regimens by ASPs, especially among the immunosuppressed, could serve as a useful strategy towards ensuring optimal use of antifungal agents.

## Abbreviations

AMC: Academic medical center
ASP: Antimicrobial stewardship program
CDB/RM: Clinical Data Base Resource Manager
CSL: Clinical service line
DOT: Days of therapy
ECMO: Extracorporeal membrane oxygenation
ELBW: Extremely low birthweight
ICD-9: International Classification of Diseases version 9
ICU: Intensive care unit
IDSA: Infectious Diseases Society of America
IFI: Invasive fungal infection
IQR: Interquartile range
LOT: Length of therapy
NICU: Neonatal intensive care unit
PD: Patient days
SD: Standard deviation
SHEA: Society for Healthcare Epidemiology of America
US: United States of America
